# Supplementary material for: Expression and phylogeny of multidrug resistance protein 2 and 4 in African white backed vulture (Gyps africanus)
Source: PeerJ. 2020 Dec 1;8:e10422. doi: 10.7717/peerj.10422 (PMC7718797; doi:10.7717/peerj.10422)
Supplement: Supplemental Information 1 [file peerj-08-10422-s001.docx]

**Expression and phylogeny of multidrug resistance protein 2 and 4 in African White Backed vulture *(Gyps africanus).***

Bono Nethathe^1, 3^, Aron Abera^2^, Vinny Naidoo^1^

Supplementary Table 1: Primers sequences used to amplify OAT1 and OAT2 gene from AWB`s kidney.

| MRPs Primers | Predicted amplicon length |
| --- | --- |
| MRP2 F(S1): CTGTGTGTCATCAGGGATTTTGTC | 2754bp |
| MRP2-IN-R(S1): ACAAGTTCAGGAATTGGGCAAAG |  |
| MRP2-IN-F(S2): CAGAGATTGGAGAGAAGGGCATT | 2550bp |
| MRP2 R(S2): AAGGTTAACAGCTCACTCAAGTCC |  |
| MRP4 F(S1): TATTGGCCATAAACGGAAGCTTGA | 2241bp |
| MRP4-IN-R(S1): GCCACCGTTAAACCTGCATAAAT |  |
| MRP4 S1: TCCTGTCCAGACCTGCATTG | To cover the sequences that were not covered by the original sequence |
| MRP4-IN- F(S2): GAACTCCCAACCTGAAGTCTGTC | 2136bp |
| MRP4 R(S2): CACTTGCAAACATTGTCACTGAGT |  |
| MRP4 S2: ATCCACAACTCGAAGTCCAGT | To cover the sequences that were not covered by the original sequence. |

F-forward primer, R- reverse primer, S1-segment 1, S2-segment 2.

Supplementary Table 2: Avian MRP2 and 4 sequences accession number used for phylogenetic analysis

| Species | Common name | MRP2 | MRP4 |
| --- | --- | --- | --- |
| Acanthisitta chloris | Rifleman | XM_009079998.1 |  |
| Anas platyrhynchos | Mallard |  | XM_021269067.1 |
| Anser cygnoides domesticus | Domestic geese | XM_013196310.1 | XM_013195781.1 |
| Apaloderma vittatum | Bar-toiled trogon |  | XM_009864407.1 |
| Aptenodytes forsteri | Emperor penguin | XM_009278032.1 |  |
| Apteryx australis mantelli | North Island Brown Kiwi |  | XM_013951288.1 |
| Aquila chrysaetos Canadensis | Golden eagle | XM_030031380.1 | XM_030036470.1 |
| Balearica regulorum gibbericeps | East-African grey crowned-crane | XM_010301460.1 |  |
| Calidris pugnax | Ruff | XM_014957963.1 | XM_014955633.1 |
| Caprimulgus carolinensis | chuck-will`s widow | XM_010175463.1 | XM_010169845.1 |
| Cariama cristata | Red-legged seriema | XM_009708646.1 | XM_009699864.1 |
| Chaetura pelagica | Chimney Swift |  | XM_010006355.1 |
| Charadrius vociferous | Killdeer | XM_009880472.1 | XM_009891916.1 |
| Chlamydotis macqueenii | Macqueen`s bustard | XM_010129192.1 |  |
| Colius striatus | Speckled mousebird |  | XM_010201078.1 |
| Columba livia | Rock Pigeon | XM_005506981.3 | XM_005505863.3 |
| Corvus brachyrhynchos | American crow | XM_017726096.1 | XM_017732634.1 |
| Cuculus canorus | Common Cuckoo |  | XM_009555936.1 |
| Egretta garzetta | Little egret | XM_009641936.1 | XM_009634600.1 |
| Eurypyga helias | Sun bittern | XM_010147273.1 | XM_010157385.1 |
| Falco cherrug | Saker falcon | XM_014276741.1 | XM_005443961.2 |
| Falco peregrinus | Peregrine falcon | XM_013300438.1 | XM_005235621.2 |
| Fulmarus glacialis | Northern Fulmar | XM_009575606.1 |  |
| Gallus gallus | Chicken | XM_015288821.1 | NM_001030819.1 |
| Gavia stellata | Red-throated loon | XM_009812133.1 |  |
| Gyps himalayensis | Himalayan Vulture |  | KX168697.1 |
| Haliaeetus leucocephalus | Bald eagle | XM_010564882.1 | XM_010578329.1 |
| Leptosomus discolor | Cuckoo roller | XM_009958793.1 |  |
| Manacus vitellinus | Golden-Collared Manakin |  | XM_018071273.1 |
| Meleagris gallopavo | Turkey | XM_010714591.2 | XM_019612046.1 |
| Melopsittacus undulates | Budgerigar |  | XM_005144756.1 |
| Nestor notabilis | Kea |  | XM_010019073.1 |
| Nipponia Nippon | Crested ibis | XM_009465232.1 | XM_009467038.1 |
| Numida meleagris | Helmeted guinea fowl | XM_021398589.1 | XM_021416653.1 |
| Opisthocomus hoazin | Stinkbird |  | XM_009942362.1 |
| Parus major | Great tit | XM_015632687.2 | XR_001520195.2 |
| Pelecanus crispus | Dalmatian pelican | XM_009485052.1 | XM_009488769.1 |
| Phaethon lepturus | White-tailed trophicbird | XM_010294140.1 |  |
| Phalacrocorax carbo | Great cormorant | XM_009500518.1 |  |
| Pygoscelis adeliae | Adelie Penguin | XM_009323318.1 | XM_009325566.1 |
| Struthio camelus australis | Southern Ostrich | XM_009677757.1 | XM_009667376.1 |
| Sturnus vulgaris | Common starling | XM_014885931.1 | XM_014881347.1 |
| Tinamus guttatus | white-throated tinamou | XM_010213115.1 | XM_010219632.1 |
| Tyto alba | Barn owl | XM_009971968.1 | XM_009963037.1 |
| Zonotrichia albicollis | White-throated sparrow | XM_014275581.1 |  |





Pane A

NTC AWB

NTC AWB

NTC AWB

NTC AWB

MRP4 S2

MRP4 S1

MRP2 S2

MRP2 S1


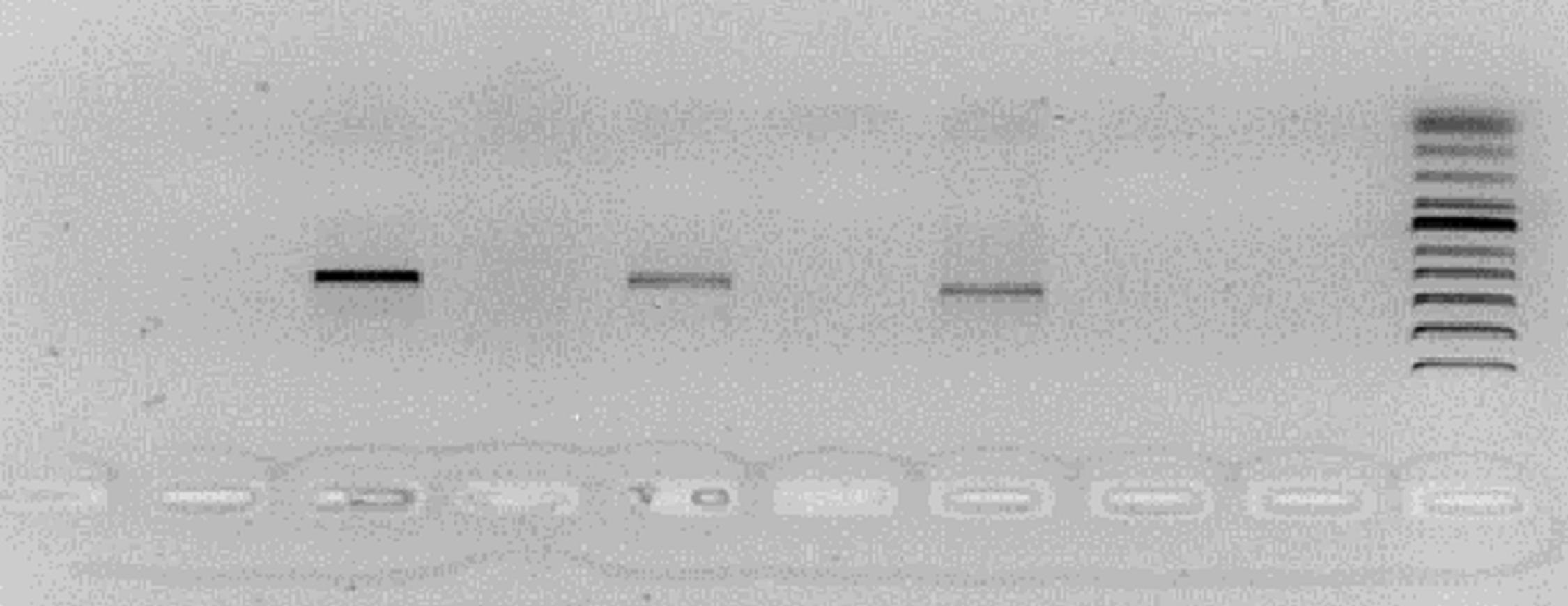


Pane B

NTC

MRP2 S1 (2574bp)

Supplementary Figure 1: Conventional PCR amplified MRP2 S2, MRP4 S1 and S2 (Pane A) and MRP2 S1 (Pane B) genes from AWB`s kidney, no product was obtained when the template was omitted, Molecular size (100bp) is indicated on the right. NTC= no template control.

MRP2_gene ------------------------------------------------------------ 0

Predicted_MRP2 tgcaatagcaccaagttaacaattaactgtgtgtcatcagggattttgtcccaacaccac 60

MRP2_gene ------------------------------------------------------------ 0

Predicted_MRP2 ttgagggtgtgtggggacttttgtcccaagcatccatccaggactcacagccaggcagag 120

MRP2_gene ---------ggagctgtcgtctcttcccccctgagccatgtcggcagccctggaggagtt 51

Predicted_MRP2 cgaggggaaggagctgtcgtctcttcccccctgagccatgtcggcagccctggaggagtt 180

***************************************************

MRP2_gene ctgtggctccgkcttttggaatgcatcctacctcactcgtccagatgccgacctgcccgt 111

Predicted_MRP2 ctgtggctccgtcttttggaatgcatcctacctcactcgtccagatgccgacctgcccgt 240

*********** ************************************************

MRP2_gene gtgcttccagcagactgtgctggtctgggtcccccttggcttcttctggattttggctcc 171

Predicted_MRP2 gtgcttccagcagactgtgctggtctgggtcccccttggcttcttctggattttggctcc 300

************************************************************

MRP2_gene atggcagctcctgcccatgtgcaaatccagagccaagaaatcatctgtgaccaaactcta 231

Predicted_MRP2 atggcagctcctgcccatgtgcaaatccagagccaagaaatcatctgtgaccaaactcta 360

************************************************************

MRP2_gene catcatcaaacaggtgctggctaccttgctgatgctgacggcagcagcggagttggcctt 291

Predicted_MRP2 catcatcaaacaggtgctggctaccttgctgatgctgacggcagcagcggagttggcctt 420

************************************************************

MRP2_gene ggcgtttgtagaggacacagagcaggaccccctgccagctgtccagtacacaaaccccag 351

Predicted_MRP2 ggcgtttgtagaggacacagagcaggaccccctgccagctgtccagtacacaaaccccag 480

************************************************************

MRP2_gene cctgtacattgccacctg------------------------------------------ 369

Predicted_MRP2 cctgtacattgccacctggctcctggtcctgctgatccatgatgcacgacgcttctgctt 540

******************

MRP2_gene ------------------------------------------------------------ 369

Predicted_MRP2 gcgcagagactcggggatacttttctgcttctggacactgtccctgctctgtgggatatt 600

MRP2_gene ---------------------------------ggcaccaatctctgacgtgccacggtt 396

Predicted_MRP2 gccattccagtcactcctccggaaagccctgcaggcaccaatctctgacgtgccacggtt 660

***************************

MRP2_gene tgtccttttcttcacctcctacgggctccagctgctgctttttcttgtctcgggcttctc 456

Predicted_MRP2 tgtccttttcttcacctcctacgggctccagctgctgctttttcttgtctcgggcttctc 720

************************************************************

MRP2_gene agacgttgccccagaaacaaaggaaatcacgaagaagaacccacaggtgacagcctcctt 516

Predicted_MRP2 agacgttgccccagaaacaaaggaaatcacgaagaagaacccacaggtgacagcctcctt 780

************************************************************

MRP2_gene cctgagctccatcacctttgaatggtacaccagcatggttttcaagggctatcgcaaacc 576

Predicted_MRP2 cctgagctccatcacctttgaatggtacaccagcatggttttcaagggctatcgcaaacc 840

************************************************************

MRP2_gene cttggagatagaggatatctgggaattgaaaggtaaagacaagacgcaggctatttatgc 636

Predicted_MRP2 cttggagatagaggatatctgggaattgaaaggtaaagacaagacgcaggctatttatgc 900

************************************************************

MRP2_gene tgttttggagaataacatgaagactgcggtgaggaaggcccaagcagaactggagaaacg 696

Predicted_MRP2 tgttttggagaataacatgaagactgcggtgaggaaggcccaagcagaactggagaaacg 960

************************************************************

MRP2_gene gaaacgcaagaaaagacgccgggaaggtgacccagaccatgggaacaacatgagcaaggc 756

Predicted_MRP2 gaaacgcaagaaaagacgccgggaaggtgacccagaccatgggaacaacatgagcaaggc 1020

************************************************************

MRP2_gene ccagagccaagacatcctggtgctggaggaaaagcagctgaagaggaagaagaaagggag 816

Predicted_MRP2 ccagagccaagacatcctggtgctggaggaaaagcagctgaagaggaagaaga-agggag 1079

***************************************************** ******

MRP2_gene acaaaggggactctggccctcacaaggatttcccccggggctggttggtgaaaaccctgt 876

Predicted_MRP2 acaaaggggactctggccctcacaaggatttcccccggggctggttggtgaaaaccctgt 1139

************************************************************

MRP2_gene gcaagaccttctggcagaacctcc------------------------------------ 900

Predicted_MRP2 gcaagaccttctggcagaacctcctgctatcggtggctttcaagctggtgcatgacggac 1199

************************

MRP2_gene ------------------------------------------------------------ 900

Predicted_MRP2 ttgtgttcgtcagcccccagctgctgaagctgctgatcgcctttgtgtcagatgaggagt 1259

MRP2_gene ------------------------------------------------------------ 900

Predicted_MRP2 cctttgcctggcaaggctatctgtatgccatcctgctcttcctgacggcactgatccagt 1319

MRP2_gene ------------------------------------------------------------ 900

Predicted_MRP2 ccctctgcctgcagcagtacttcagcttgtgcttccagcttggcataaatgtgcgtgcca 1379

MRP2_gene ------------------------------------------------------------ 900

Predicted_MRP2 gtctcattgctgccatctacaagaaggcactcaccatgtccagtgccacccgcaaggagt 1439

MRP2_gene ------------------------------------------------------------ 900

Predicted_MRP2 ccacggtgggagagactgtgaatctgatgtcagctgatgcccagaggttcatggacacgg 1499

MRP2_gene ------------------------------------------------------------ 900

Predicted_MRP2 ccaacttcgttcaccagctgtggtcatcccccctgcaaattatcctgtccattgtcttcc 1559

MRP2_gene ---------------------------------------cagttatggtgctgctcatcc 921

Predicted_MRP2 tctggggagagctgggcccctctgttctggctggcatcgcagttatggtgctgctcatcc 1619

*********************

MRP2_gene cccataaatgggttcctggttgccaaggccaaaaccatccaggtgaggaacatgaagaac 981

Predicted_MRP2 -ccataaatgggttcctggttgccaaggccaaaaccatccaggtgaggaacatgaagaac 1678

***********************************************************

MRP2_gene aaggatgaacgcatgaaaataatgagtgaaatcctcaatggaatcaagatcctgaagctt 1041

Predicted_MRP2 aaggatgaacgcatgaaaataatgagtgaaatcctcaatggaatcaagatcctgaagctt 1738

************************************************************

MRP2_gene tttgcctgggagccctcatttgagaagcgagtcaatgagatccgggcacatgagctcaag 1101

Predicted_MRP2 tttgcctgggagccctcatttgagaagcgagtcaatgagatccgggcacgtgagctcaag 1798

************************************************* **********

MRP2_gene gacttggtgaacttcagttacctgcagtcaatctctatcttcgtgttcacgtgtgcgccc 1161

Predicted_MRP2 gacttggtgaacttcagttacctgcagtcaatctctatcttcgtgttcacgtgtgcgccc 1858

************************************************************

MRP2_gene ttcctggtctccttggccagctttgctgtttacatgctggtggatgagaacaacatcctg 1221

Predicted_MRP2 ttcctggtctccttggccagctttgctgtttacatgctggtggatgagaacaacatcctg 1918

************************************************************

MRP2_gene gatgcacagaaagcctttactgccatctcccttttcaacgtgctgcgcttccccatggcc 1281

Predicted_MRP2 gatgcacagaaagcctttactgccatctcccttttcaacgtgctgcgcttccccatggcc 1978

************************************************************

MRP2_gene atgctgcccttggtcctttcttccttggtgcagaccaacgtgtcgactgcgaggctggag 1341

Predicted_MRP2 atgctgcccttggtcctttcttccttggtgcagaccaacgtgtcgactgcgaggctggag 2038

************************************************************

MRP2_gene cgctacctgggcagagaagacctggacacctcggctatccaccacaaccccattgcaggc 1401

Predicted_MRP2 cgctacctgggcagagaagacctggacacctcggctatccaccacaaccccattgcag-- 2096

**********************************************************

MRP2_gene aggcagcgctgtgcgtttctcggaggccacctttgcctgggagcaggacggcaatgctgc 1461

Predicted_MRP2 --gcagcgctgtgcgtttctcggaggccacctttgcctgggagcaggacggcaatgctgc 2154

**********************************************************

MRP2_gene gataagagatgtcaccctggacatcgcacctgggagcctggtggccgtggtgggggctgt 1521

Predicted_MRP2 gataagagatgtcaccctggacatcgcacctgggagcctggtggccgtggtgggggctgt 2214

************************************************************

MRP2_gene gggctcaggcaaatcttcgctggtgtcagccatgctcggggagatggagaatatcaaggg 1581

Predicted_MRP2 gggctcaggcaaatcttcgctggtgtcagccatgctcggggagatggagaatatcaaggg 2274

************************************************************

MRP2_gene acacatcaacatccagggctccctggcctatgtaccccagcaggcctggatccagaatgc 1641

Predicted_MRP2 acacatcaacatccagggctccctggcctatgtaccccagcaggcctggatccagaatgc 2334

************************************************************

MRP2_gene cacactgaaagacaacatcctttttgggtcagaactggatgaagccaggtatcagcaggt 1701

Predicted_MRP2 cacactgaaagacaacatcctttttgggtcagaactggatgaagccaggtatcagcaggt 2394

************************************************************

MRP2_gene catcaaggcctgcgccctccttccagacctggaactgctgcctkygggtgrccagacaga 1761

Predicted_MRP2 catcaaggcctgcgccctccttccagacctggaactgctgcctgcgggtgaccagacaga 2454

******************************************* ***** *********

MRP2_gene gattggagagaagggcattaacctgagcgggggccagaagcagc---------------- 1805

Predicted_MRP2 gattggagagaagggcattaacctgagcgggggccagaagcagcgagtcagcctggcccg 2514

********************************************

MRP2_gene ------------------------------------------------------------ 1805

Predicted_MRP2 ggcagtgtacagcaacgcagacatctacatcctggatgaccccctgtctgccgtggatgc 2574

MRP2_gene ------------------------------------------------------------ 1805

Predicted_MRP2 tcatgtcggcaagtacctcttcgagcatgtgctggggccaaaagggctgctgcaaaagaa 2634

MRP2_gene ------------------------------------------------------------ 1805

Predicted_MRP2 gacacggatcttggtgacgcacagtatcagtttcctgccccaggtcgataacatcgtggt 2694

MRP2_gene ------------------------------------------------------------ 1805

Predicted_MRP2 gctggtggcaggaacagtgtctgagcatggctcctacagcaccctgcttgcaaacagggg 2754

MRP2_gene ------------------------------------------------------------ 1805

Predicted_MRP2 ggcctttgcccaattcctgaacttgtacggcagccaggaggaggatgcttcagagaagaa 2814

MRP2_gene ------------------------------------------------------------ 1805

Predicted_MRP2 taccacagctgttgctttagctggggatgaagagcagggtgatgaagacattgagccttg 2874

MRP2_gene ------------------------------------------------------------ 1805

Predicted_MRP2 tgtggaggagggtcctgatgatgtggtgaccatgaccctgaagcgcgacgccagcatccg 2934

MRP2_gene ------------------------------------------------------------ 1805

Predicted_MRP2 tcagagagagttcagtcgcagccttagtaaaagcagcaccaattcctggaagaaggccca 2994

MRP2_gene ------------------------------------------------------------ 1805

Predicted_MRP2 ggaggagccccccaagaagctgaaaggccagcagctgattgagaaagaagctgtggaaac 3054

MRP2_gene -----aggtgaagttctccatgtacctgcggtacctgcatgccgttggcttgtggtattc 1860

Predicted_MRP2 cggcaaggtgaagttctccatgtacctgcggtacctgcatgccgttggcttgtggtattc 3114

*******************************************************

MRP2_gene tttctgggttgccatgggctacgttggacagtacgtcgccttcgtggggactaacctgtg 1920

Predicted_MRP2 tttctgggttgccatgggctacgttggacagtacgtcgccttcgtggggactaacctgtg 3174

************************************************************

MRP2_gene gctcagtgcctggactgacgatgcgcagcactacctgaaccagacctatcccacagagca 1980

Predicted_MRP2 gctcagtgcctggactgacgatgcgcagcactacctgaaccagacctatcccacagagca 3234

************************************************************

MRP2_gene gcgggacctgcggatcggtgtctttggggcactgggggtgtcacaagctctcttcctgct 2040

Predicted_MRP2 gcgggacctgcggatcggtgtctttggggcactgggagtgtcacaagctctcttcctgct 3294

************************************ ***********************

MRP2_gene ccttgcaaccctcctgtctgctcgtggtgccatgcgagcctcgcgggttatgcatcagca 2100

Predicted_MRP2 ccttgcaaccctcctgtctgctcgtggtgccatgcgagcctcgcgggttatgcatcagca 3354

************************************************************

MRP2_gene actgctcagcaacatcctgcgtgtgcccatgagcttttttgacacaaccccgactggccg 2160

Predicted_MRP2 actgctcagcaacatcctgcgtgtgcccatgagcttttttgacacaaccccgactggccg 3414

************************************************************

MRP2_gene cattgtgaataggtttgccaaggacatcttcacgatagatgagaccattcctatgtcctt 2220

Predicted_MRP2 cattgtgaataggtttgccaaggacatcttcacgatagatgagaccattcccatgtcctt 3474

*************************************************** ********

MRP2_gene ccgcagctggctctcctgtttcatggccatcattagcacattgctcatgatctccctggc 2280

Predicted_MRP2 ccgcagctggctctcctgtttcatggccatcattagcacattgctcatgatctccctggc 3534

************************************************************

MRP2_gene caccccattcttcactctcgttatcattcccttgagcatcttctactattttgtgctgcg 2340

Predicted_MRP2 caccccattcttcactctcgttatcattcccttgagcatcttctactattttgtgctgcg 3594

************************************************************

MRP2_gene cttctatgtctccacatcacgccagctaaggcgtctggactctgtaactaggtctcccat 2400

Predicted_MRP2 cttctatgtctccacatcacgccagctaaggcgtctggactctgtaactaggtctcccat 3654

************************************************************

MRP2_gene ctactcccactttggcgagacagtgtcagggctttctgtgatccgtgccttcggacacca 2460

Predicted_MRP2 ctactcccactttggcgagacagtgtcagggctttctgtgatccgtgccttcggacacca 3714

************************************************************

MRP2_gene agaacgattcctgcagcagaatgagagcaccatggacgtcaatcaaaaaagtgtttactc 2520

Predicted_MRP2 agaacgattcctgcagcagaatgagagcaccatggacgtcaatcaaaaaagtgtttactc 3774

************************************************************

MRP2_gene ctggatagtctcaaataggtggctggccatccgtctggagttcgttgggagcctggtggt 2580

Predicted_MRP2 ctggatagtctcaaataggtggctggccatccgtctggagttcgttgggagcctggtggt 3834

************************************************************

MRP2_gene cttcttctctgcgcttctagctgtgatttcaaagggcactttggagggcggcatcgtggg 2640

Predicted_MRP2 cttcttctctgcgcttctagctgtgatttcaaagggcactttggagggcggcatcgtggg 3894

************************************************************

MRP2_gene tctttctgtctcctctgccctcaatgtgacccagacactgaactggctggtgcggacgtc 2700

Predicted_MRP2 tctttctgtctcctctgccctcaatgtgacccagacactgaactggctggtgcggacgtc 3954

************************************************************

MRP2_gene ttcggagctggagacaaacattgtggctgtggagcgggtacatgagtacacgaaggtgaa 2760

Predicted_MRP2 ttcggagctggagacaaacattgtggctgtggagcgggtacatgagtacacgaaggtgaa 4014

************************************************************

MRP2_gene gaatgaggctccgtgggtgacagaaaagcgtccaccccatggctggcccagcaaaggtga 2820

Predicted_MRP2 gaatgaggctccgtgggtgacagaaaagcgtccaccccatggctggcccagcaaaggtga 4074

************************************************************

MRP2_gene gatccagtttgttgactacaaagttcgttaccgacctgaactggagctggttcttcaggg 2880

Predicted_MRP2 gatccagtttgttgactacaaagttcgttaccgacctgaactggagctggttcttcaggg 4134

************************************************************

MRP2_gene gatcacctgcaatattgggagcacggagaaggttggggttgtgggccggactggggctgg 2940

Predicted_MRP2 gatcacctgcaatattgggagcacggagaaggttggggttgtgggccggactggggctgg 4194

************************************************************

MRP2_gene aaaatcttccctcaccaactgcctcttccgggtgctggaggccgctggagggacgatcat 3000

Predicted_MRP2 aaaatcttccctcaccaactgcctcttccgggtgctggaggccgctggagggacgatcat 4254

************************************************************

MRP2_gene catcgacgaagtggatatagcaacgatcggcctccatgacctgcgccagaacctcaccat 3060

Predicted_MRP2 catcgacgaggtggatatagcaacgatcggcctccatgacctgcgccagaacctcaccat 4314

********* **************************************************

MRP2_gene catccctcaggaccccgtgctctttactggcaccctgcggatgaacctggatccctttga 3120

Predicted_MRP2 catccctcaggaccccgtgctctttactggcaccctgcggatgaacctggatccctttga 4374

************************************************************

MRP2_gene ccagtacatggatgaggaggtctggaaggcccttgagctggcccacctgaagacatatgt 3180

Predicted_MRP2 ccagtacatggatgaggaggtctggaaggcccttgagctggcccacctgaagacatatgt 4434

************************************************************

MRP2_gene gcaagaccttcccgaggggctgctgcatcttgtgagcgaggcgggggagaacctgagtgt 3240

Predicted_MRP2 gcaagaccttcccgaggggctgctgcatcttgtgagcgaggcgggggagaacctgagtgt 4494

************************************************************

MRP2_gene tgggcagaggcagctggtgtgcctggcccgggcncctccttcgcaaagccaagatcctca 3300

Predicted_MRP2 tgggcagaggcagctggtgtgcctggcccgggc-cctccttcgcaaagccaagatcctca 4553

********************************* **************************

MRP2_gene tcctggacgaagcgacagcagccgtagat------------------------------- 3329

Predicted_MRP2 tcctggacgaagcgacagcagccgtagatctagaaactgatcatttaatccagacaacga 4613

*****************************

MRP2_gene ------------------------------------------------------------ 3329

Predicted_MRP2 tccggagtgagtttgctgactgcactgtccttactattgcccaccgcctccacaccatca 4673

MRP2_gene ------------------------------------------------------------ 3329

Predicted_MRP2 tggacagcaacagggtgatggtgctgcaggctgggaggattgtggaatacgacagccctg 4733

MRP2_gene ------------------------------------------------------------ 3329

Predicted_MRP2 aggagctgctcaagaagcacggtgtcttctccgcaatggcaaaggacgctggcatcacga 4793

MRP2_gene ------------------------------------------------------------ 3329

Predicted_MRP2 atatagaaaccactgtgctgtaggtggagcagagcagtgtgcgggtgtgtgcgttggcag 4853

MRP2_gene ------------------------------------------------------------ 3329

Predicted_MRP2 ctccctcccactggcactcaccaagcaggcagcagcttctccctgctgccgggctgccca 4913

MRP2_gene ------------------------------------------------------------ 3329

Predicted_MRP2 ggaaattctctctgcagctgggaagcagagagagtggcttctctggccaggacagaggat 4973

MRP2_gene ------------------------------------------------------------ 3329

Predicted_MRP2 ctggacttgagtgagctgttaaccttgctaccccaccctgcttgctgtgcgcatgagggt 5033

MRP2_gene ------------------------------------------------------------ 3329

Predicted_MRP2 ctggagctgcataatttattccagtatagaggtgaaaagtctgccatgggagaccatgac 5093

MRP2_gene ------------------------------------------------------------ 3329

Predicted_MRP2 ccccgtgggggtcttagtttttgtacttcaccatgccaggggaacctagctgagatatgc 5153

MRP2_gene ----------------------------------------------------------- 3329

Predicted_MRP2 tttagcactacggaatgaaatttacagtttaatctaagagtgttataaacttttgtaac 5212

Supplementary Figure 2: Alignment of MRP2 gene of AWB vulture using Sanger and next generation sequencing revealing similarity of 99.76% with clusta omega software. MRP2_gene = Sanger; Predicted_MRP2 = NGS

MRP4_gene ------------------------------------------------------------ 0

Predicted_MRP4 ggtggttgaatcctttatttattattggccataaacggaagcttgaagaagatgatatgt 60

MRP4_gene --------------GAAGATTCCTCAGAGAAGCTTGGAGAGGAATTGCAGTGGTACTGGG 46

Predicted_MRP4 ataaagtgctgccagaagattcctcagagaagcttggagaggaattgcagtggtactggg 120

**********************************************

MRP4_gene MTAAARAGGTGCAAAWAGCAWAAAAKAGAGGAAAAACGCCACGTTTAACAAAAGCCATTA 106

Predicted_MRP4 ataaagaggtgcaaaaagcaaaaaagagaggaaaaacgccacatttaacaaaagccatta 180

**** ********* **** **** **************** *****************

MRP4_gene TTCTTTGTTACTGGAAATCCTATTTAKTTTTTGGAATTTTCACAATGATTGAGGAAACCC 166

Predicted_MRP4 ttctttgttactggaaatcctatttagtttttggaattttcacaatgattgaggaaaccc 240

************************** *********************************

MRP4_gene TCAAAATAATTCAGCCAATATTTTTGGGAAAAATTATTAATTATTTTGAAAACTATGAT- 225

Predicted_MRP4 tcaaaataattcagccaatatttttgggaaaaattattaattattttgaaaactatgatt 300

***********************************************************

MRP4_gene CCTCAGATGAGGTAGCTTTGAATTTTGCATATTTCTACGCAGCTGCTCTGTCTGTGTGCA 285

Predicted_MRP4 cctcagatgaggtagctttgaattttgcatatttctacgcagctgctctgtctgtgtgca 360

************************************************************

MRP4_gene CGCTTATTCTAGCTATAATGCACCACTTATACTTCTATCATGTACAGCGGGCTGGCATGA 345

Predicted_MRP4 cgcttattctagctataatgcaccacttatacttctatcatgtacagcgggctggcatga 420

************************************************************

MRP4_gene AGCTGAGGGTAGCTATGTGTCACATGATTTATCGRAAGGCACTTCGTCTCAGTAACGTAG 405

Predicted_MRP4 agctgagggtagctatgtgtcacatgatttatcggaaggcacttcgtctcagtaacgtag 480

********************************** *************************

MRP4_gene CTATGGCAAAAACTACCACTGGKCAAATAGTGAATCTTCTGTCAAATGATGTGAACAAAT 465

Predicted_MRP4 ctatggcaaaaactaccactggtcaaatagtgaatcttctgtcaaatgatgtgaacaaat 540

********************** *************************************

MRP4_gene TTGATCAGGTAACAATCTTCTTGCACTTCTTGTGGGCTGGACCAATTCAAGCTGTARCAG 525

Predicted_MRP4 ttgatcaggtaacaatcttcttgcacttcttgtgggctggaccaattcaagctgtagcag 600

******************************************************** ***

MRP4_gene TAACAGTACTTCTCTGGATGGAGATAGGCCCATCATGTCTTGCAGGAATGGCAGYTCTGA 585

Predicted_MRP4 taacagtacttctctggatggagataggcccatcatgtcttgcaggaatggcagttctga 660

****************************************************** *****

MRP4_gene TTATTCTTCTTCCTGTCCAGACCTGCATTGGGAGGCTTTTTTCTTCCCTAAGAAGCAAGA 645

Predicted_MRP4 ttattcttcttcctgtccagacctgcattgggaggcttttttcttccctaagaagcaaga 720

************************************************************

MRP4_gene CAGCTGCCTTARCAGATGTCAGGATTAGGACCATGAATGAAGTCATAAGTGGTATGAAGA 705

Predicted_MRP4 cagctgccttaacagatgtcaggattaggaccatgaatgaagtcataagtggtatgaaga 780

*********** ************************************************

MRP4_gene TAAATAAAAGATGTATGCTTGGGAAAAATCATTTGCGGAACTTGTGAATGGTTTAAGAAG 765

Predicted_MRP4 taat--aaagatgtatgcttgggaaaaatcatttgcggaacttgtgaatggtttaagaag 838

*** ******************************************************

MRP4_gene GAAGGAGATTTGCCATGGTTATGAAAAAGCTCCTACCTTCGAGGACTGAACTTAACCTCA 825

Predicted_MRP4 gaaggagat-tgccatggttatgaa-aagctcctaccttcgaggactgaacttagcctca 896

********* *************** **************************** *****

MRP4_gene TTTTTTGTGGCAAGCAAAATAACAGTGTTCATGACTTTCATGGCATATGTACTACTTGGC 885

Predicted_MRP4 ttttttgtggcaagcaaaataacagtgttcatgactttcatggcatatgtactacttggc 956

************************************************************

MRP4_gene AATGTTATCTCTGCAAGTCGGGTGTTTGTTGCAGTGTCCCTGTATGGTGCAGTAAGACTG 945

Predicted_MRP4 aatgttatctctgcaagtcgggtgtttgttgcagtgtccctgtatggtgcagtaagactg 1016

************************************************************

MRP4_gene ACAGTAACTCTGTTCTTCCCTTCGGCTATTGAGAGAGTATCCGAGGCAGTGGTTAGCATA 1005

Predicted_MRP4 acagtaactctgttcttcccttcggctattgagagagtatccgaggcagtggttagcata 1076

************************************************************

MRP4_gene CGACGAATCAAGAACTTTCTGATACTTGATGAGATCTCACCCTTCAAGCCACAACTGCAT 1065

Predicted_MRP4 cgacgaatcaagaactttctgatacttgatgagatctcacccttcaagccacaactgcat 1136

************************************************************

MRP4_gene GGTAATAATGAGAATGTCATTCTTCATGTTCAGGATTTGACTTGCTATTGGGATAAGAGT 1125

Predicted_MRP4 ggtaataatgagaatgtcattcttcatgttcaggatttgacttgctattgggataagagt 1196

************************************************************

MRP4_gene TTAGAAAGCCCAGCACTTCAACAACTTTCATTTACTGTCAGACGAGGGGAATTATTGGCT 1185

Predicted_MRP4 ttagaaagcccagcacttcaacaactttcatttactgtcagacgaggggaattattggct 1256

************************************************************

MRP4_gene GTGATTGGTCCTGTAGGAGCTGGCAAATCTTCACTCTTAAGTGCTGTGCTTGGTGAGCTA 1245

Predicted_MRP4 gtgattggtcctgtaggagctggcaaatcttcactcttaagtgctgtgcttggtgagcta 1316

************************************************************

MRP4_gene CCTAAAGACAAAGGTTTGATAAATGTTACTGGAAGAATTGCCTATGTTTCTCAGCAGCCT 1305

Predicted_MRP4 cctaaagacaaaggtttgataaatgttactggaagaattgcctatgtttctcagcagcct 1376

************************************************************

MRP4_gene TGGGTGTTTTCTGGTACAGTAAGAAGTAATATACTGTTTGACAAGGNAATATGAAAAAGA 1365

Predicted_MRP4 tgggtgttttctggtacagtaagaagtaatatactgtttgacaagg-aatatgaaaaaga 1435

********************************************** *************

MRP4_gene AAAATACGAAAAAGTTTTAAAAGTCTGTGCTCTTAAAAAGGACTTGGAATTATTAGCRAA 1425

Predicted_MRP4 aaaatacgaaaaagttttaaaagtctgtgctcttaaaaaggacttggaattattagcaaa 1495

********************************************************* **

MRP4_gene TGGTGACCTAACAGTAATAGGAGATCGTGGAGCTACGCTGAGTGGGGGACAGAAAGCCCG 1485

Predicted_MRP4 tggtgacctaacagtaataggagatcgtggagctacgctgagtgggggacagaaagcccg 1555

************************************************************

MRP4_gene TGTAAATCTGGCCAGAGCTGTGTATCAAGATGCAGACATCTATCTTTTTGGATGATCCAC 1545

Predicted_MRP4 tgtaaatctggccagagctgtgtatcaagatgcagacatctatctt-ttggatgatccac 1614

********************************************** *************

MRP4_gene TGAGTGCAGTAGATGCTGAAGTTGGAAGACATTTGTTTGAAAAATGTATTTGTCAGGCCT 1605

Predicted_MRP4 tgagtgcagtagatgctgaagttggaagacatttgtttgaaaaatgtatttgtcaggcct 1674

************************************************************

MRP4_gene TWCATCAGAAGATCTCTGTTTTGGTTACTCACCAGTTGCAGTATCTCCGTGCTGCAAATC 1665

Predicted_MRP4 tacatcagaagatctctgttttggttactcaccagttgcagtatctccgtgctgcaaatc 1734

* **********************************************************

MRP4_gene AGATTCTAATTTTAAAAGATGGTAAAATGGTGGGGAAAGGTACCTATTCAGAGTTCCTGA 1725

Predicted_MRP4 agattctaattttaaaagatggtaaaatggtggggaaaggtacctattcagagttcctga 1794

************************************************************

MRP4_gene GATCTGGCATCGACTTTGCTTCCCTTTTGAAAAAAGATGAGGAGGTAGAACAGCCGTCAG 1785

Predicted_MRP4 gatctggcatcgactttgcttcccttttgaaaaaagatgaggaggtagaacagctgtcag 1854

****************************************************** *****

MRP4_gene TTCCAGGAACTCCCAACCTGAAGTCTGTCCGGAGCCGAACCTTCTCAGAGTCCTCTGTCT 1845

Predicted_MRP4 ttccaggaactcccaacctgaagtctgtccggagccgaaccttctcagagtcctctgtct 1914

************************************************************

MRP4_gene GGTCCCAGGATTCTTCTGCCCACTCACAGAAAGATGGAGCAGTGGAGCAACCACCTGCTG 1905

Predicted_MRP4 ggtcccaggattcttctgcccactcacagaaagatggagcagtggagcaaccacctgctg 1974

************************************************************

MRP4_gene AAAACGCACTGGCTGCAGTGCCAGAGGAGAGTCGCTCTGAGGGAAAAATAAACTTTAAGG 1965

Predicted_MRP4 aaaacgcactggctgcagtgccagaggagagtcgctctgagggaaaaataaactttaagg 2034

************************************************************

MRP4_gene TTTACAGAAAATATTTCACTGCAGGAGCAAACTACTTTGTGATTTTCATACTTCTAGTAT 2025

Predicted_MRP4 tttacagaaaatatttcactgcaggagcaaactactttgtgattttcatacttctagtat 2094

************************************************************

MRP4_gene TCAATATTTTGGCACAGGTGGCATACGTGCTCCAGGACTGGTGGCTTTCTTACTGGGCAA 2085

Predicted_MRP4 tcaatattttggcacaggtggcatacgtgctccaggactggtggctttcttactgggcaa 2154

************************************************************

MRP4_gene ATCATCAAGAAAAGTTGAACGTCACAACAAATGGAAATAATGGAGCAAATGAGAGTGAAC 2145

Predicted_MRP4 atcatcaagaaaagttgaacgtcacaacaaatggaaataatggagcaaatgagagtgaac 2214

************************************************************

MRP4_gene ATCTAGACCTTAACTTTTATTTGGGAATTTATGCAGGTTTAACGGTGGCTACAATACTGT 2205

Predicted_MRP4 atctagaccttaacttttatttgggaatttatgcaggtttaacggtggctacaatactgt 2274

************************************************************

MRP4_gene TTGGCATAGTAAGAAGTCTTTTGGTGTTTCAAGTTCTTGTTAATTCTGGTCAGACTTTGC 2265

Predicted_MRP4 ttggcatagtaagaagtcttttggtgtttcaagttcttgttaattctggtcagactttgc 2334

************************************************************

MRP4_gene ACAACAAAATGTTTCAATCCATTTTGAAAGCTCCCGTCTTGTTTTTTGACAGAAATCCTA 2325

Predicted_MRP4 acaacaaaatgtttcaatccattttgaaagctcccgtcttgttttttgacagaaatccta 2394

************************************************************

MRP4_gene TAGGAAGAATCTTAAATCGTTTCTCCAAAGATATTGGCCACCTGGATGACTTGCTTCCAT 2385

Predicted_MRP4 taggaagaatcttaaatcgtttctccaaagatattggccacctggatgacttgcttccat 2454

************************************************************

MRP4_gene TGACATTTTTGGACTTCATGCAGACTCTCCTACAGATTTTTGGTGTGGTGGCTGTGGCTG 2445

Predicted_MRP4 tgacatttttggacttcatgcagactctcctacagatttttggtgtggtggctgtggctg 2514

************************************************************

MRP4_gene TGGCAGTGATTCCTTGGATACTCCTCCCCTTAATTCCACTATTTATTCTTTTCATTTTCC 2505

Predicted_MRP4 tggcagtgattccttggatactcctccccttaattccactatttattcttttcattttcc 2574

************************************************************

MRP4_gene TTCGACGATATTTCTTAGACACTTCAAGAGATATTAAACGTCTAGAATCCACAACTCGAA 2565

Predicted_MRP4 ttcgacgatatttcttagacacttcaagagatattaaacgtctagaatccacaactcgaa 2634

************************************************************

MRP4_gene GTCCAGTGTTCTCCCACTTGTCGTCATCCCTCCAGGGACTTTGGACTATTCGGGCTTTGA 2625

Predicted_MRP4 gtccagtgttctcccacttgtcgtcatccctccagggactttggactattcgggctttga 2694

************************************************************

MRP4_gene AAGCAGAGGAAAGATTTCAAAAATTATTTGATGCACACCAAGACCTCCACTCAGAGGCCT 2685

Predicted_MRP4 aagcagaggaaagatttcaaaaattatttgatgcacaccaagacctccactcagaggcct 2754

************************************************************

MRP4_gene GGTTTCTATTTTTGACGACCTCGAGGTGGTTTGCTGTGCGTCTGGATGCCATCTGTGCCA 2745

Predicted_MRP4 ggtttctatttttgacgacctcgaggtggtttgctgtgcgtctggatgccatctgtgcca 2814

************************************************************

MRP4_gene TTTTTGTTATAGTGGTTGCTTTTGGTTCCCTGCTTCTCKCCAAGACTTTGAATGCAGGGC 2805

Predicted_MRP4 tttttgttatagtggttgcttttggttccctgcttctctccaagactttgaatgcagggc 2874

************************************** *********************

MRP4_gene AGGTTGGTTTGGCACTATCCTATGCAATCACCCTCATGGGAACATTCCAGTGGGGTGTTA 2865

Predicted_MRP4 aggttggtttggcactatcctatgcaatcaccctcatgggaacattccagtggggtgtta 2934

************************************************************

MRP4_gene GACAAAGTGCTGAAGTTGAAAACCTGATGATATCAGTAGAAAGAGTAATGGAATACACAG 2925

Predicted_MRP4 gacaaagtgctgaagttgaaaacctgatgatatcagtagaaagagtaatggaatacacag 2994

************************************************************

MRP4_gene AACTTGAAAAAGAAGCTCCTTGGGAGACCAACAAGCATCCACCACCTGAATGGCCAAGCC 2985

Predicted_MRP4 aacttgaaaaagaagctccttgggagaccaacaagcatccaccacctgaatggccaagcc 3054

************************************************************

MRP4_gene AAGGAATGATAGCATTTGAAAATGTTAACTTCACTTACAGTCTAGATGGACCTTTGGTGT 3045

Predicted_MRP4 aaggaatgatagcatttgaaaatgttaacttcacttacagtctagatggacctttggtgt 3114

************************************************************

MRP4_gene TAAGACATTTGTCTGTTTTAATTAAACCAAAAGAAAAGGTTGGAATAGTGGGAAGAACTG 3105

Predicted_MRP4 taagacatttgtctgttttaattaaaccaaaagaaaaggttggaatagtgggaagaactg 3174

************************************************************

MRP4_gene GAGCTGGGAAAAGCTCTCTGATAGCAGCCCTCTTTCGCTTGGCGGAACCCGAAGGAAGGA 3165

Predicted_MRP4 gagctgggaaaagctctctgatagcagccctcttccgcttggcggaacccgaaggaagga 3234

********************************** *************************

MRP4_gene TTTGGATTGATAAGTACTTGACGTCAGAGCTAGGACTCCATGACTTGCGGAAGAAAATTT 3225

Predicted_MRP4 tttggattgataagtacttgacgtcagagctaggactccatgacttgcggaagaaaattt 3294

************************************************************

MRP4_gene CAATTATACCTCAGGAGCCTGTTTTATTCACTGGAACTATGAGGAAAAACTTAGATCCTT 3285

Predicted_MRP4 caattatacctcaggagcctgttttattcactggaactatgaggaaaaacttagatcctt 3354

************************************************************

MRP4_gene TCAATGAATACACTGATGAGGAGCTGTGGAATGCCTTGGAAGAGGTGCAACTGAAGGAGG 3345

Predicted_MRP4 tcaatgaatacactgatgaggagctgtggaatgccttggaagaggtgcaactgaaggagg 3414

************************************************************

MRP4_gene TTGTGGAAGATCTACCTAATAAAATGGAGATGCAGCTGGCAGAAATCTGGGTCTAATTTT 3405

Predicted_MRP4 ttgtggaagatctacctaataaaatggagatgcagctggcaga-atctgggtctaatttt 3473

******************************************* ****************

MRP4_gene AGTGTTGGTCAGAGACAGCTGGTGTGTCTTGCCAGAGCAGTTCTAAAAAAAAAATCGGAT 3465

Predicted_MRP4 agtgttggtcagagacagctggtgtgtcttgccagagcagttctaaaa-aaaaatcggat 3532

************************************************ ***********

MRP4_gene CCTTATCATTGATGAAGCAACAGCAAATGTGGACCCAAGAACAGATGAGTTYATTCAAAA 3525

Predicted_MRP4 ccttatcattgatgaagcaacagcaaatgtggacccaagaacagatgagtttattcaaaa 3592

*************************************************** ********

MRP4_gene GACGATCCGTGAAAAGTTTGCTCACTGCACAGTGCTGACCATTGCACACCGCTTGAACAC 3585

Predicted_MRP4 gacgatccgtgaaaagtttgctcactgcacagtgctgaccattgcacaccgcttgaacac 3652

************************************************************

MRP4_gene CATTATTGACAGTGACAGGATTATGGTTTTAGATGAAGGAAGAGTGAAAGAATATGGTGA 3645

Predicted_MRP4 cattattgacagtgacaggattatggttttagatgaaggaagagtgaaagaatatggtga 3712

************************************************************

MRP4_gene ACCTTACATTTTGCTGCAAGAACAAGATGGCTTGTTTTACAAAATGGTGCAACAAGTGGG 3705

Predicted_MRP4 accttacattttgctgcaagaacaagatggcttgttttacaaaatggtgcaacaagtggg 3772

************************************************************

MRP4_gene CAAGACTGAAGCAGCTTCTYTGATTGAAACAGCAAAACGGGTGTACTTCAGTAAGAATTA 3765

Predicted_MRP4 caagactgaagcagcttctctgattgaaacagcaaaacgggtgtacttcagtaagaatta 3832

******************* ****************************************

MRP4_gene CCCAGAAGTTGTTCAGAATGGTCAACTTGCCACAGACTCCTCCTTGGATCCTTCCTCAGG 3825

Predicted_MRP4 cccagaagttgttcagaatggtcaacttgccacagactcctccttggatccttcctcagg 3892

************************************************************

MRP4_gene ATTAKGCATAACCGAAACTGCACTGTGATTCCTAATAA---------------------- 3863

Predicted_MRP4 attatgcataaccgaaactgcactgtgattcctaataaccttaactgttttccattgaat 3952

**** *********************************

MRP4_gene ------------------------------------------------------------ 3863

Predicted_MRP4 gtaaacctgagatcatctaaactcagtgacaatgtttgcaagtgtcagcaggagaggaaa 4012

MRP4_gene ------------------------------------------------- 3863

Predicted_MRP4 gggagggggcgattctttgcactggacatccttcctatttaatactgag 4061

Supplementary Figure 3: Alignment of MRP4 gene of AWB vulture using Sanger and next generation sequencing revealing similarity of 99.43% with clusta omega software. . MRP4_gene = Sanger; Predicted_MRP4 = NGS

Extra TMD with 5 TMH


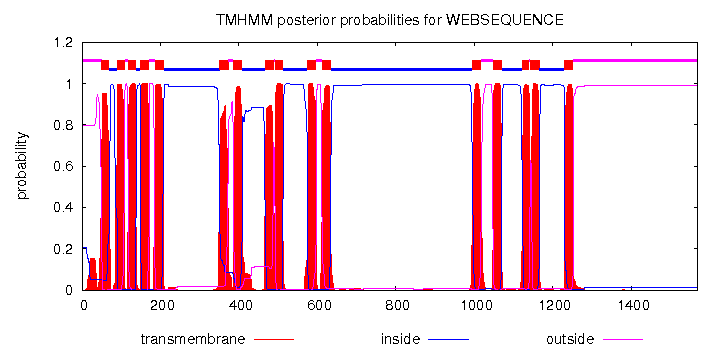


Pane A


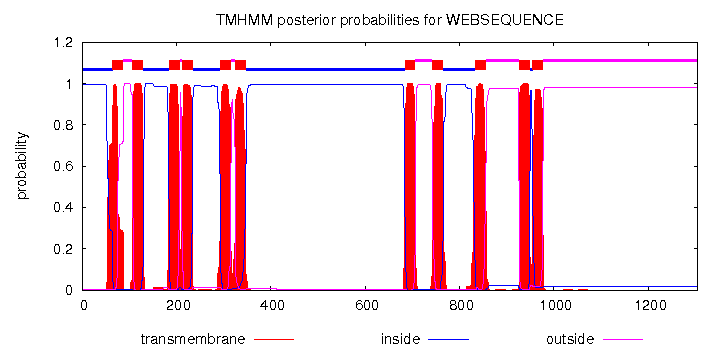


Pane B

Supplementary Figure 4: Prediction of transmembrane helices in A) MRP2 revealing the presence of 16 TMH and B) MRP4 showing the presence of 11 TMH.
