# Supplementary material for: Expression and phylogeny of multidrug resistance protein 2 and 4 in African white backed vulture (Gyps africanus)
Source: PeerJ. 2020 Dec 1;8:e10422. doi: 10.7717/peerj.10422 (PMC7718797; doi:10.7717/peerj.10422)
Supplement: Supplemental Information 2 [file peerj-08-10422-s002.pdf]

> Predicted Vulture MRP2 (MN691108).

tgcaatagcaccaagttaacaattaactgtgtgtcatcagggattttgtcccaacaccacttgagggtgtgtggggacttttgtcccaagcatccat
ccaggactcacagccaggcagagcgaggggaaggagctgtcgtctcttccccctgagccatgtcggcagccctggaggagttctgtggctccg
tcttttggaatgcatcctacctcactcgtccagatgccgacctgcccgtgtgcttccagcagactgtgctggtctgggtccccttggcttcttctgg
attttggctccatggcagctcctgcccatgtgcaaatccagagccaagaaatcatctgtgaccaaactctacatcatcaaacaggtgctggctac
cttgctgatgctgacggcagcagcggagttggccttggcgtttgtagaggacacagagcaggacccctgccagctgtccagtacacaaacccc
agcctgtacattgccacctggctcctggtcctgctgatccatgatgcacgacgcttctgcttgcgcagagactcggggatacttttctgcttctggac
actgtccctgctctgtgggatattgccattccagtcactcctccggaaagccctgcaggcaccaatctctgacgtgccacggtttgtccttttcttca
cctcctacgggctccagctgctgctttttcttgtctcgggcttctcagacgttgccccagaaacaaaggaaatcacgaagaagaacccacaggtg
acagcctccttcctgagctccatcacctttgaatggtacaccagcatggttttcaagggctatcgcaaacccttggagatagaggatatctgggaa
ttgaaaggtaaagacaagacgcaggctatttatgctgttttggagaataacatgaagactgcggtgaggaaggcccaagcagaactggagaaa
cggaaacgcaagaaaagacgccgggaaggtgacccagaccatgggaacaacatgagcaaggcccagagccaagacatcctggtgctggag
gaaaagcagctgaagaggaagaagaagggagacaaaggggactctggccctcacaaggatttcccccggggctggttggtgaaaaccctgtg
caagaccttctggcagaacctcctgctatcggtggctttcaagctggtgcatgacggacttgtgttcgtcagcccccagctgctgaagctgctgatc
gcctttgtgtcagatgaggagtcctttgcctggcaaggctatctgtatgccatcctgctcttcctgacggcactgatccagtccctctgcctgcagca
gtacttcagcttgtgcttccagcttggcataaatgtgcgtgccagtctcattgctgccatctacaagaaggcactcaccatgtccagtgccacccgc
aaggagtccacggtgggagagactgtgaatctgatgtcagctgatgcccagaggttcatggacacggccaacttcgttcaccagctgtggtcatc
ccccctgcaaattatcctgtccattgtcttcctctggggagagctgggcccctctgttctggctggcatcgcagttatggtgctgctcatccccataa
atgggttcctggttgccaaggccaaaaccatccaggtgaggaacatgaagaacaaggatgaacgcatgaaaataatgagtgaaatcctcaatg
gaatcaagatcctgaagctttttgcctgggagccctcatttgagaagcgagtcaatgagatccgggcacgtgagctcaaggacttggtgaacttc
agttacctgcagtcaatctctatcttcgtgttcacgtgtgcgcccttcctggtctccttggccagctttgctgtttacatgctggtggatgagaacaac
atcctggatgcacagaaagcctttactgccatctcccttttcaacgtgctgcgcttccccatggccatgctgcccttggtcctttcttccttggtgcag
accaacgtgtcgactgcgaggctggagcgctacctgggcagagaagacctggacacctcggctatccaccacaaccccattgcaggcagcgct
gtgcgtttctcggaggccacctttgcctgggagcaggacggcaatgctgcgataagagatgtcaccctggacatcgcacctgggagcctggtgg
ccgtggtgggggctgtgggctcaggcaaatcttcgctggtgtcagccatgctcggggagatggagaatatcaagggacacatcaacatccaggg
ctccctggcctatgtaccccagcaggcctggatccagaatgccacactgaaagacaacatcctttttgggtcagaactggatgaagccaggtatc
agcaggtcatcaaggcctgcgccctccttccagacctggaactgctgcctgcgggtgaccagacagagattggagagaagggcattaacctgag
cggggccagaagcagcgagtcagcctggcccgggcagtgtacagcaacgcagacatctacatcctggatgacccccctgtctgccgtggatgct
catgtcggcaagtacctcttcgagcatgtgctggggccaaaagggctgctgcaaaagaagacacggatcttggtgacgcacagtatcagtttcct
gccccaggtcgataacatcgtggtgctggtggcaggaacagtgtctgagcatggctcctacagcaccctgcttgcaaacagggggcctttgccc
aattcctgaacttgtacggcagccaggaggaggatgcttcagagaagaataccacagctgttgctttagctggggatgaagagcagggtgatga
agacattgagccttgtgtggaggagggtcctgatgatgtggtgaccatgaccctgaagcgcgacgccagcatccgtcagagagagttcagtcgc
agccttagtaaaagcagcaccaattcctggaagaaggcccaggaggagcccccaagaagctgaaaggccagcagctgattgagaaagaag
ctgtggaaaccggcaaggtgaagttctccatgtacctgcggtacctgcatgccgttggcttgtggtattctttctgggttgccatgggctacgttgg
acagtacgtcgccttcgtggggactaacctgtggctcagtgcctggactgacgatgcgcagcactacctgaaccagacctatcccacagagcag
cgggacctgcggatcggtgtctttggggcactgggagtgtcacaagctctcttcctgctccttgcaaccctcctgtctgctcgtggtgccatgcgag
cctcgcgggttatgcatcagcaactgctcagcaacatcctgcgtgtgcccatgagctttttgacacaaccccgactggccgcattgtgaataggt
ttgccaaggacatcttcacgatagatgagaccattcccatgtccttccgcagctggctctcctgtttcatggccatcattagcacattgctcatgatc
tccctggccaccccattcttcactctcgttatcattcccttgagcatcttctactattttgtgctgcgcttctatgtctccacatcacgccagctaaggc
gtctggactctgtaactaggtctcccatctactcccactttggcgagacagtgtcagggctttctgtgatccgtgccttcggacaccaagaacgatt
cctgcagcagaatgagagcaccatggacgtcaatcaaaaaagtgtttactcctggatagtctcaaataggtggctggccatccgtctggagttcg
ttgggagcctggtggtcttcttctctgcgcttctagctgtgatttcaaagggcactttggagggcggcatcgtgggtctttctgtctcctctgccctca
atgtgacccagacactgaactggctggtgcggacgtcttcggagctggagacaaacattgtggctgtggagcgggtacatgagtacacgaaggt
gaagaatgaggctccgtgggtgacagaaaagcgtccaccccatggctggcccagcaaaggtgagatccagtttgttgactacaaagttcgttac
cgacctgaactggagctggttcttcaggggatcacctgcaatattgggagcacggagaaggttggggttgtgggccggactggggctggaaaat
cttccctcaccaactgcctcttccgggtgctggaggccgctggagggacgatcatcatcgacgaggtggatatagcaacgatcggcctccatgac
ctgcgccagaacctcaccatcatccctcaggaccccgtgctctttactggcaccctgcggatgaacctggatccctttgaccagtacatggatgag

gaggtctggaaggcccttgagctggcccacctgaagacatatgtgcaagaccttcccgaggggctgctgcatcttgtgagcgaggcgggggaga
acctgagtgttgggcagaggcagctggtgtgcctggcccgggccctccttcgcaaagccaagatcctcatcctggacgaagcgacagcagccgt
agatctagaaactgatcatttaatccagacaacgatccggagtgagtttgctgactgcactgtccttactattgcccaccgcctccacaccatcat
ggacagcaacagggtgatggtgctgcaggctgggaggattgtggaatacgacagccctgaggagctgctcaagaagcacggtgtcttctccgc
aatggcaaaggacgctggcatcacgaatatagaaaccactgtgctgtaggtggagcagagcagtgtgcgggtgtgtgcgttggcagctccctcc
cactggcactcaccaagcaggcagcagcttctccctgctgccgggctgcccaggaaattctctctgcagctgggaagcagagagagtggcttctc
tggccaggacagaggatctggacttgagtgagctgttaaccttgctaccccaccctgcttgctgtgcgcatgagggtctggagctgcataatttat
tccagtatagaggtgaaaagtctgccatgggagaccatgacccccgtgggggtcttagtttttgtacttcaccatgccaggggaacctagctgag
atatgctttagcactacggaatgaaatttacagtttaatctaagagtgttataaacttttgtaac

> Predicted Vulture MRP4 (MN691109).

ggtggttgaatcctttatttattattggccataaacggaagcttgaagaagatgatatgtataaagtgctgccagaagattcctcagagaagcttg
gagaggaattgcagtggtactgggataaagaggtgcaaaaagcaaaaaagagaggaaaaacgccacatttaacaaaagccattattctttgtt
actggaaatcctatttagtttttggaattttcacaatgattgaggaaaccctcaaaataattcagccaatatttttgggaaaaattattaattatttt
gaaaactatgattcctcagatgaggtagctttgaattttgcatatttctacgcagctgctctgtctgtgtgcacgcttattctagctataatgcacca
cttatacttctatcatgtacagcgggctggcatgaagctgagggtagctatgtgtcacatgatttatcggaaggcacttcgtctcagtaacgtagct
atggcaaaaactaccactggtcaaatagtgaatcttctgtcaaatgatgtgaacaaatttgatcaggtaacaatcttcttgcacttcttgtgggctg
gaccaattcaagctgtagcagtaacagtacttctctggatggagataggcccatcatgtcttgcaggaatggcagttctgattattcttcttcctgt
ccagacctgcattgggaggctttttttcttccctaagaagcaagacagctgccttaacagatgtcaggattaggaccatgaatgaagtcataagtg
gtatgaagataataaagatgtatgcttgggaaaaatcatttgcggaacttgtgaatggtttaagaaggaaggagattgccatggttatgaaaag
ctcctaccttcgaggactgaacttagcctcatttttttgtggcaagcaaaataacagtgttcatgactttcatggcatatgtactacttggcaatgtta
tctctgcaagtcgggtgtttgttgcagtgtccctgtatggtgcagtaagactgacagtaactctgttcttcccttcggctattgagagagtatccgag
gcagtggttagcatacgacgaatcaagaactttctgatacttgatgagatctcacccttcaagccacaactgcatggtaataatgagaatgtcatt
cttcatgttcaggatttgacttgctattgggataagagtttagaaagcccagcacttcaacaactttcatttactgtcagacgaggggaattattgg
ctgtgattggtcctgtaggagctggcaaatcttcactcttaagtgctgtgcttggtgagctacctaaagacaaaggtttgataaatgttactggaa
gaattgcctatgtttctcagcagccttgggtgttttctggtacagtaagaagtaatatactgtttgacaaggaatatgaaaaagaaaaatacgaa
aaagttttaaaagtctgtgctcttaaaaaggacttggaattattagcaaatggtgacctaacagtaataggagatcgtggagctacgctgagtgg
gggacagaaagcccgtgtaaatctggccagagctgtgtatcaagatgcagacatctatcttttggatgatccactgagtgcagtagatgctgaag
ttggaagacatttgtttgaaaaatgtatttgtcaggccttacatcagaagatctctgttttggttactcaccagttgcagtatctccgtgctgcaaat
cagattctaattttaaaagatggtaaaatggtggggaaaggtacctattcagagttcctgagatctggcatcgactttgcttcccttttgaaaaaa
gatgaggaggtagaacagctgtcagttccaggaactcccaacctgaagtctgtccggagccgaaccttctcagagtcctctgtctggtcccagga
ttcttctgcccactcacagaaagatggagcagtggagcaaccacctgctgaaaacgcactggctgcagtgccagaggagagtcgctctgaggg
aaaaataaactttaaggtttacagaaaatatttcactgcaggagcaaactactttgtgattttcatacttctagtattcaatattttggcacaggtg
gcatacgtgctccaggactggtggctttcttactgggcaaatcatcaagaaaagttgaacgtcacaacaaatggaaataatggagcaaatgaga
gtgaacatctagaccttaacttttatttgggaatttatgcaggtttaacggtggctacaatactgtttggcatagtaagaagtcttttggtgtttcaa
gttcttgttaattctggtcagactttgcacaacaaaatgtttcaatccattttgaaagctcccgtcttgtttttttgacagaaatcctataggaagaat
cttaaatcgtttctccaaagatattggccacctggatgacttgcttccattgacatttttggacttcatgcagactctcctacagatttttggtgtggt
ggctgtggctgtggcagtgattccttggatactcctccccttaattccactatttattcttttcattttccttcgacgatatttcttagacacttcaaga
gatattaaacgtctagaatccacaactcgaagtccagtgttctcccacttgtcgtcatccctccagggactttggactattcgggctttgaaagcag
aggaaagatttcaaaaattatttgatgcacaccaagacctccactcagaggcctggtttctatttttgacgacctcgaggtggtttgctgtgcgtct
ggatgccatctgtgccatttttgttatagtggttgcttttggttccctgcttctctccaagactttgaatgcagggcaggttggtttggcactatcctat
gcaatcaccctcatgggaacattccagtggggtgttagacaaagtgctgaagttgaaaacctgatgatatcagtagaaagagtaatggaataca
cagaacttgaaaaagaagctccttgggagaccaacaagcatccaccacctgaatggccaagccaaggaatgatagcatttgaaaatgttaact
tcacttacagtctagatggacctttggtgttaagacatttgtctgttttaattaaaccaaaagaaaaggttggaatagtgggaagaactggagctg
ggaaaagctctctgatagcagccctcttccgcttggcggaacccgaaggaaggatttggattgataagtacttgacgtcagagctaggactccat
gacttgcggaagaaaatttcaattatacctcaggagcctgttttattcactggaactatgaggaaaaacttagatcctttcaatgaatacactgat

gaggagctgtggaatgccttggaagaggtgcaactgaaggaggttgtggaagatctacctaataaaatggagatgcagctggcagaatctggg
tctaattttagtgttggtcagagacagctggtgtgtcttgccagagcagttctaaaaaaaaatcggatccttatcattgatgaagcaacagcaaat
gtggacccaagaacagatgagtttattcaaaagacgatccgtgaaaagtttgctcactgcacagtgctgaccattgcacaccgcttgaacaccat
tattgacagtgacaggattatggttttagatgaaggaagagtgaaagaatatggtgaaccttacattttgctgcaagaacaagatggcttgtttta
caaaatggtgcaacaagtgggcaagactgaagcagcttctctgattgaaacagcaaaacgggtgtacttcagtaagaattacccagaagttgtt
cagaatggtcaacttgccacagactcctccttggatccttcctcaggattatgcataaccgaaactgcactgtgattcctaataaccttaactgtttt
ccattgaatgtaaacctgagatcatctaaactcagtgacaatgtttgcaagtgtcagcaggagaggaaagggagggggcgattctttgcactgg
acatccttcctatttaatactgag

SRA accession number: PRJNA560189 is too big to attached however when the article is ready for publication it will be released to public by NCBI.
